# Laboratory Analysis of VOC Emissions from Structural Materials in Wildland–Urban Interface Fires

**DOI:** 10.1021/acs.est.5c11276

**Published:** 2026-02-20

**Authors:** William Dresser, Kevin Ridgway, Anna Helfrich, Christian L’Orange, Shantanu Jathar, Joost de Gouw

**Affiliations:** † Department of Chemistry, 1877 University of Colorado, Boulder, Colorado 80309, United States; ‡ Cooperative Institute for Research in Environmental Science, University of Colorado/NOAA, Boulder, Colorado 80309, United States; § Department of Mechanical Engineering, 3447 Colorado State University, Fort Collins, Colorado 80523, United States

**Keywords:** volatile organic compounds, wildland−urban interface, urban wildfires, emission factors, air pollution

## Abstract

The wildland–urban interface (WUI) has grown in recent decades at the same time as wildfires have expanded in range and scope. Fires at the WUI are therefore more common, and structural materials make up more of the wildfire fuel mass. While emissions from biomass fires are fairly well understood, volatile organic compound (VOC) emissions from structural fires are less constrained. In this study, we perform measurements of VOC emissions from small-scale laboratory burns of 18 different structural materials across 78 experiments using a Vocus proton-transfer reaction time-of-flight mass spectrometer (PTR-ToF-MS) to better understand these unique emissions. We calculate emission factors for 73 VOCs across all materials, including aromatics and polycyclic aromatic hydrocarbons (PAHs). We compare the emissions from both flaming and pyrolysis, which separate the processes of direct release of VOCs from combustion formation. Mass spectra comparisons were used to qualitatively highlight high-emission compounds across materials and identify notable emissions (e.g., nylon monomers) and potential tracers (e.g., halogen species) for WUI fires. Using these data, the emissions from a whole-house fire were compared with those from an equivalent mass of wood, and we found that some aromatic and nitrile species may be suitable WUI fire tracers.

## Graphical abstract

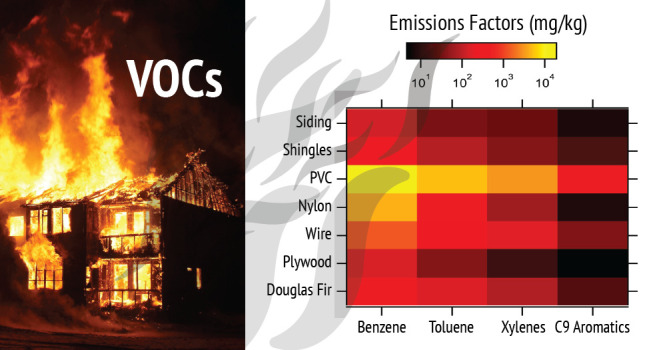

## Introduction

The wildland–urban interface (WUI) is defined as the area where human development meets the natural landscape, and its extent has increased over the past several decades in the U.S.^1^ This development has happened at the same time as the range and frequency of wildfires have increased across the U.S., especially in the western United States.^2–4^ Recent fires at the WUI have led to significant loss of life and structures.^5,6^ Beyond these immediate concerns, WUI fire emissions represent an increased air quality concern as they occur closer to communities.^7^ Additionally, the man-made materials that are burned are potentially associated with higher air toxics emissions.^8–10^ Smoke exposure not only occurs during WUI fires, but can also linger for weeks in indoor environments after a fire.^11–13^

Wildfire emissions are concerning for both their immediate health impacts and their ability to react in the atmosphere to create both ozone and secondary organic aerosols (SOA).^8,14^ The volatile organic compound (VOC) emissions from biomass wildfires have been studied both in the field and constrained through laboratory experiments.^15–18^ This work has established that emissions can vary depending on the biomass material burned and the type of combustion. Previous work has indicated that pyrolysis is a key process in the release of VOCs from biomass burning.^19^ Pyrolysis is the thermal decomposition of materials, which can occur at the high temperatures (250–800 °C) present in wildfires, while there is limited oxygen. Pyrolysis produces gases that combust in the flames or can escape the flames and be released into the air.

The emissions from the burning of structural materials are less well understood. A few studies have looked at select VOC emissions, mainly in simulated laboratory settings.^20–22^ Work by Holder et al. synthesized this previous work into an overview of emissions from the burning of structural materials, with an emphasis on aromatic and polycyclic aromatic hydrocarbons (PAHs).^23^ They showed consistent enhancements in burning emissions from structural materials compared with biomass materials. There still exist questions, though, on the broader VOC composition from the burning of specific materials. The potential exists that more unusual compounds could have very high emissions from the burning of select materials and could serve as tracer compounds in field measurements of VOCs.

In this work, we measured VOC emissions from the burning of small amounts of common structural materials. Emission factors for the flaming and pyrolysis of 18 different materials were quantified and are reported here for 73 VOC compounds. The burning spectra for different materials were compared against a biomass spectrum to search for compounds that could act as tracers in the interpretation of the WUI fire field data. We also created estimated profiles for emissions from an average home and compared them to similar amounts of biomass material to identify differences in emissions and evaluate suitable WUI fire tracers.

## Methods

### Experimental Setup

The flaming/pyrolysis setup is described in detail in Ridgway et al.,^24^ but a short description will be given here. Materials were weighed before each experiment and placed in a basket attached to a counterweight to measure mass loss throughout the experiment. The basket was lowered into a circular furnace, which was heated to a set temperature monitored with a temperature probe placed in the oven. For pyrolysis experiments, the circular furnace was heated, and sufficient N_2_ was introduced to keep O_2_ levels low enough to prevent autoignition. For flaming experiments, the oven was turned off, and a H_2_ burner was used to create the heat and combustion. Temperatures varied by experiment, particularly for flaming experiments, with values around ∼400 °C for pyrolysis experiments and ∼300–700 °C for flaming.^24^

Emissions from the flaming/pyrolysis experiments were drawn up in a fume hood at a flow of ∼4 m^3^ min^–1^ with a flow of ∼1.5 SLPM redirected for the measurements presented here. A dilution was needed in the measured air to avoid saturating the instrument. This was done by adding UHP N_2_ at a flow of 1–1.3 SLPM from the sample line. To determine the dilution without having the sample flow go through a flow meter, we measured CO_2_ in the sample line with an Li-820 CO_2_ Gas Analyzer (LI-COR). This allowed us to quantify the dilution using a ratio of CO_2_ measurements from the sample line against a CO_2_ measurement in the main hood using a Siemens Ultramat/Oxymat 6 (SIEMENS). A linear fit between measurement values from these two instruments was made to correct for the dilution in each experiment. The total sampling line from the hood to the instrument measurement consisted of ∼3.75 m of Teflon tubing. While this length of sampling line did not affect the response time for measurements at the start of the burn, the tube length meant that there were longer inlet delay times for some less volatile compounds after the experiments. Most compounds had delay times on the order of five min, while others were longer, on the order of tens of minutes. Blank experiments were conducted in both modes to correct for detection of any VOCs not derived from the fuels or potentially from the residual emissions within the experimental setup.

### Material Overview

Nineteen unique materials were selected in the experiments that best account for the prevalence of materials in structures, particularly in the U.S. West, local availability, and in consultation with construction managers.^24,25^ We were not able to run measurements on one of the materials, so values for 18 are reported here. The materials can be divided into nine broad categories,^25^ i.e., lumber, processed wood, roofing, insulation, siding, carpet, plumbing/plastic, electrical, and flooring. The specific materials were Douglas Fir (DF), Southern Yellow Pine (SYP), Japanese Sugi (JS), Oriented Strand Board (OSB), Medium-Density Fiberboard (MDF), Plywood, Cellulose, Extruded Polystyrene (XPS), Polyurethane Foam (PUF), Polyvinyl Chloride (PVC), Chlorinated Polyvinyl Chloride (CPVC), Asphalt Shingles, Nylon Carpet, Triexta Carpet, Polyester Carpet, Cement Fiber Siding, Luxury Vinyl Plank (LVP), and Electrical Wire. Materials were cut into small homogeneous units of around 32–82 cm^3^ and a mass range between 5 and 120 g for the experiments. The fuel summary is given in Table S1. In this work, the word “synthetic” is used to refer to materials outside of the lumber and processed wood categories.

### VOC Measurements

A Vocus (Tofwerk) proton-transfer-reaction time-of-flight mass spectrometer (PTR-ToF-MS) was used for measurements of VOCs. This instrument allows for the detection of a broad range of VOCs, with the notable exception of alkanes. An instrument overview is given by Krechmer et al.^26^ For these experiments, the reduced electric field strength (E/N) in the reactor was ∼160 Td, with daily calibrations and background measurements performed between each experiment to correct for instrument drift. Backgrounds were taken by passing ambient air through a catalytic zero air generator (Tofwerk AG) and overflowing the Vocus inlet for several minutes between experiments. Calibrations were performed with a gas mixture containing 12 VOCs (Table S2) to allow for direct quantification of those species. Sensitivity and error for these compounds were calculated directly using daily multipoint calibration curves. Further calibration factors were calculated using approximations developed by Sekimoto et al., based on the relationship between sensitivity and the proton-transfer reaction coefficient (*k*_PTR_).^27^ Daily calibration values had an average error of 20 ± 20% across all experiments, with an additional error of 5% from correcting for the CO_2_ dilution. Data workup was done in software developed by Jensen et al. to allow for automation of background subtraction and calibration.^28^ A list of 73 VOCs was compiled for quantification based on interest and availability of data for calibration calculations.

### Emission Factor Calculations

Emission factors (EFs) for each VOC were calculated using methods outlined by Ridgway et al.^24^ This method creates EFs for each VOC relative to the sum of measured carbon in the smoke through combined measurements of CO_2_, CO, CH_4_, OC, EC, and the sum of the VOCs. This method then acts as a proxy for the emissions of VOCs relative to the amount of fuel carbon emitted for each material burned as opposed to fuel mass. This method inherently accounts for the burned fraction of the fuel but can be hard to use for fuels with low CO_2_ emissions (see below).

First, the measurements were corrected for average blank experiment measurements done in the relevant mode (flaming, pyrolysis). Then, we corrected for the dilution factor measured for each individual experiment and applied the calculated sensitivity values to get concentrations in units of ppb for each VOC. These ppb values can then be converted into ppm and used to calculate EF values using eq 1:

1 $${\mathrm{EF}}_{s}=\frac{C_{s}\left(\mathrm{ppm}\right)\times 10^{-6}\cdot \frac{P}{RT}\cdot {\mathrm{MW}}_{s}\cdot f_{C}}{\left(d_{{\mathrm{CO}}_{2}}+d_{\mathrm{CO}}+d_{{\mathrm{CH}}_{4}}+d_{\mathrm{VOC}}\right)\times 10^{-6}\cdot \frac{P}{RT}\cdot \mathrm{AW}\cdot d_{\mathrm{OC}}+d_{\mathrm{EC}}}$$

where EF_s_ is the value in g/g for species s; *C*_s_(ppm) is the concentration of species s in ppm; MW_s_ is the molecular weight of species s in g/mol; *f*_C_ is the carbon fraction of the specific fuel; *d*_CO_2__ is the enhancement in CO_2_ in ppm; *d*_CO_ is the enhancement in CO in ppm; *d*_CH_4__ is the enhancement in CH_4_ in ppm; *d*_VOC_ is the total emitted VOCs in ppmC; AW is the atomic weight of carbon in g/mol; *d*_OC_ is the enhancement in organic carbon (OC) in g/m^3^; and *d*_EC_ is the enhancement in elemental carbon (EC) in g/m^3^. Measurements of the parameters outlined in eq 1 were obtained from Ridgway et al.^24^ For several experiments, specifically for synthetic materials, there were no quantifiable emissions of CO_2_ and CO. In these cases, an approximation equation based on the mass lost during the burn was used for the EF calculations (eq 2):

2 $${\mathrm{EF}}_{s}=\frac{C_{s}\left(g/m^{3}\right)}{\frac{\Delta M}{V\cdot t}}$$

where EF is the value in g/g for species s; C_s_(g/m^3^) is the concentration of species s in g/m^3^; Δ*M* is the mass loss for the fuel in grams; *V* is the airflow into the duct in m^3^/min; and *t* is the time for the experiment in minutes. Errors in the parameters for both equations were between ∼1 and 2%, taken from Ridgway et al., and incorporated into the final EF error values.^24^ The agreement between the two methods is within a factor of ∼2 and is shown for a set of VOCs in Figure S1.

The EF values calculated here were compared to values derived from other VOC measurements made during the study using evacuated canisters and 2,4-dinitrophenylhydrazine (DNPH) cartridges, and analyzed offline by GC-MS (Shimadzu GC-17A/QP5050A). Individual samples were collected over the entire length of the burn. The offline measurements were sensitive to alkane species, which the PTR method is not, but PTR allows for broad detection of oxygenated, alkenes, and generally functionalized molecules. Results presented in this work relied on online measurements capturing an overall time series across the whole burn. We compared the overlapping species detected by both methods. The agreement between the two methods is shown for a subset of VOCs in Figure S2, which gives an indication of the accuracy of the results.

## Results and Discussion

An example of the data from experimental burns of Douglas Fir (DF) lumber and PVC under flaming conditions for a subset of VOCs is shown in Figure 1. For Douglas Fir, the fuel was lowered into the oven at 11:09:40, at which time the VOC concentrations rose within seconds. The variability in the emissions over time is evident, especially under flaming conditions, where the combustion varies as the fuel collapses within the basket. The biomass burning tracers show the highest concentrations in DF, which is in contrast to the experiment for PVC, where aromatics have much higher emission concentrations. These real-time data allow for investigation of how the emissions might change even within the course of a burn, which others have investigated and would be worth considering in future work; however, we will not be focusing on that here.^19^ The gray line indicates the point in the experiment when the fuel had been consumed and emissions decreased. The time frame for each experiment started when the basket entered the oven and ended when the collection on the collaborators’ filters was complete (in Figure 1 shown by black dashed lines at 11:39).^24^ Data were collected for a period of time after the material was consumed to account for smoke moving through the hood and the delay time of VOCs moving through the sampling lines. We integrated the VOC time series over this period relative to the initial value before the burn to derive a total amount for the corresponding VOC. Because the aim was for VOC measurements that corresponded to collaborators’ measurements, this meant some compound concentrations were cut off before they could return to background levels, leading to a minor underestimation. However, this allowed for a direct comparison between the real-time and offline samples. The concentrations of VOCs are insightful to understand emissions, but in order to compare to other measurements and better estimate real-world burning conditions, we use these concentrations to calculate EFs, as discussed in Methods.

**Figure 1:**
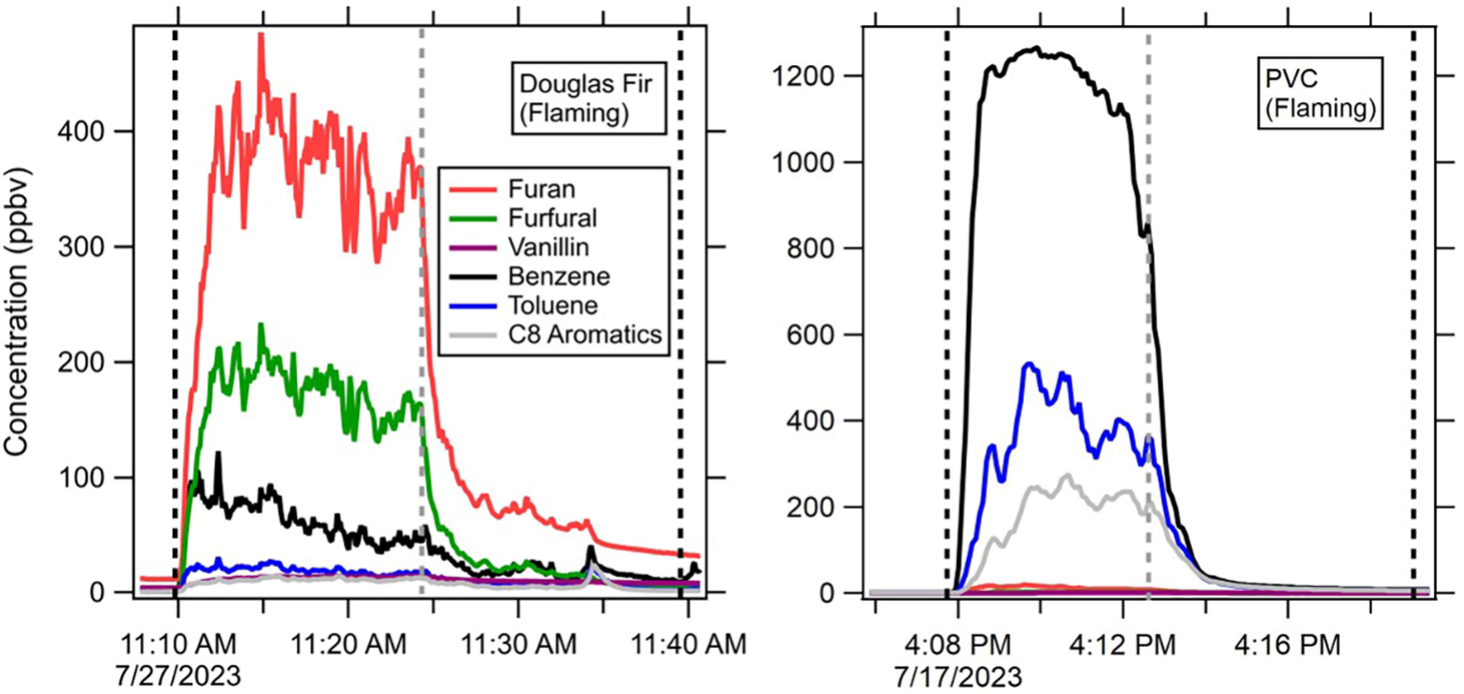
Example of the time series for selected VOCs from flaming experiments of Douglas Fir lumber and PVC. The shown VOCs represent several biomass burning tracer compounds (furan, furfural, vanillin) and aromatic species (benzene, toluene, and C8 aromatics). Dashed black lines indicate the start and end point of the time period used to calculate emissions for this experiment. The dashed gray line shows the time when the emissions dropped as the fuel was consumed.

### Emission Factors

Figure 2 summarizes the data on the calculated flaming emissions factor for several subsets of VOCs across different fuels. The color values indicate the value of the flaming EFs and are on a log scale in units of mg/kg. Figure 2A shows values for several single-ring aromatics from C6–C9. The results show that emission differences between lumber and processed wood are fairly minimal, all on the order of 10–100s mg/kg. There are orders of magnitude higher values in the synthetic materials, particularly the plastics (PVC, CPVC) and insulation materials (XPS, PUF), with values of up to 17800 ± 3700 mg/kg for benzene. Figure 2B includes several biomass burning VOC tracers, which show fairly consistent emissions across all materials, with slightly higher values for materials that contain wood. It should be noted that acetonitrile, a common biomass tracer, does not show significant differences across the material types, with most values on the order of 10–100 mg/kg. Figure 2C highlights EF values for a subset of polycyclic aromatic hydrocarbons (PAHs). These are notable for their negative health impacts, and those listed here are designated as EPA hazardous air pollutants.^29^ For all materials, we found the highest EF values for indene and naphthalene compared to other PAHs, but again we saw enhancements for several materials, specifically PVC and XPS, with values on the order of 1000 mg/kg. Another notable difference is the enhancement in higher mass PAH compounds among the synthetic materials, creating a distinct profile from biomass materials. For quantified sulfur species, we note high values of dimethyl sulfide (DMS), 1350 ± 240 mg/kg, in the cellulose insulation material. CPVC has a notably high EF value for chlorotoluene, 332 mg/kg, and XPS also shows high values for methyl chloride and ethyl chloride, likely linked to chlorinated compounds introduced in the manufacturing process.^30^ EF values and concentrations for the complete list of VOCs quantified from both flaming and pyrolysis experiments are given in Tables S3 and S4. We observed EF variability within repeat experiments around a factor of ∼2–3, which agrees with what has been seen in other work and reflects the complex nature of capturing consistent results across multiple experiments.^16,24^ For materials where we had repeat experiments, combined mean values are reported where relevant in the figures. During several experiments, there were issues with the measurement of some parameters in eq 1 and those do not have EF values but do have concentrations.

**Figure 2:**
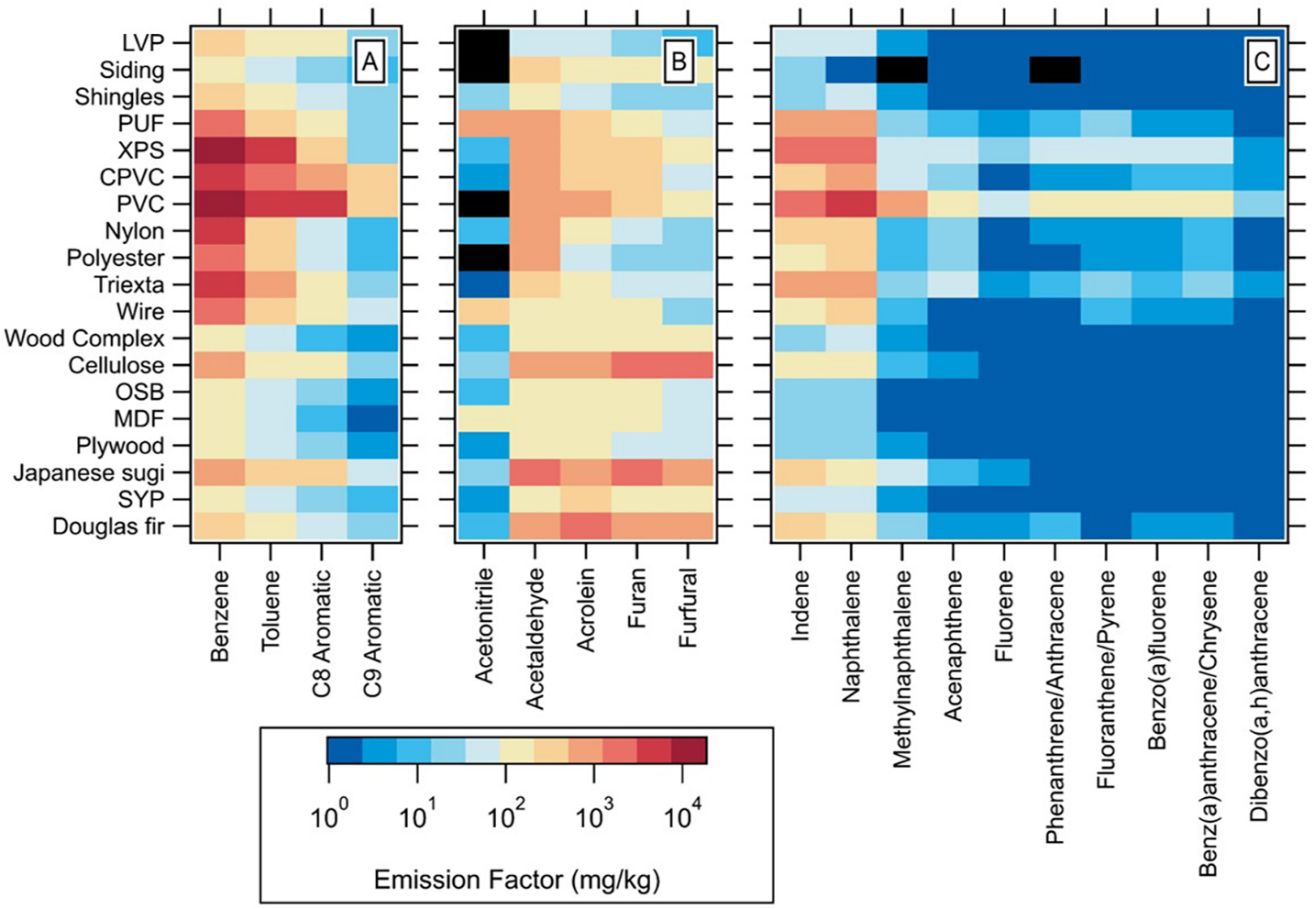
Flaming emission factors for several VOCs across the tested materials: (A) several aromatic VOCs; (B) nitrogen and biomass burning (BB) tracer molecules; (C) PAH compounds. Values are shown on a log scale. Black squares indicate values that were not discernible above the background.

### Flaming versus Pyrolysis

As discussed in Methods, experiments were done under both flaming and pyrolysis conditions due to the different natures of VOC emissions in each process. Pyrolysis is an anoxic thermal degradation process and generally has emissions that are composed of the molecules that make up a material or fragments thereof. For homogeneous materials, the pyrolysis process can lead to emissions of both monomers and polymer compounds, such as nylon monomers and dimers from carpet. In flames, VOCs produced by pyrolysis combust at least partially to CO_2_ and as a result, the overall EFs are lower. This is indeed what is seen for biomass materials in Figure 3A. This panel shows the difference between average EF values across PAH compounds in the lumber materials for flaming and pyrolysis: pyrolysis EFs are higher by an order of magnitude. Figure 3B shows the comparison of flaming and pyrolysis EF values for all quantified VOCs, which shows a consistent factor between the two processes. Evidently, the flames combust different VOCs by the same factor. There are some outliers, notably benzene, styrene, and benzonitrile, which show values closer to the one-to-one line, indicating some formation in the flames offsetting their removal.

**Figure 3:**
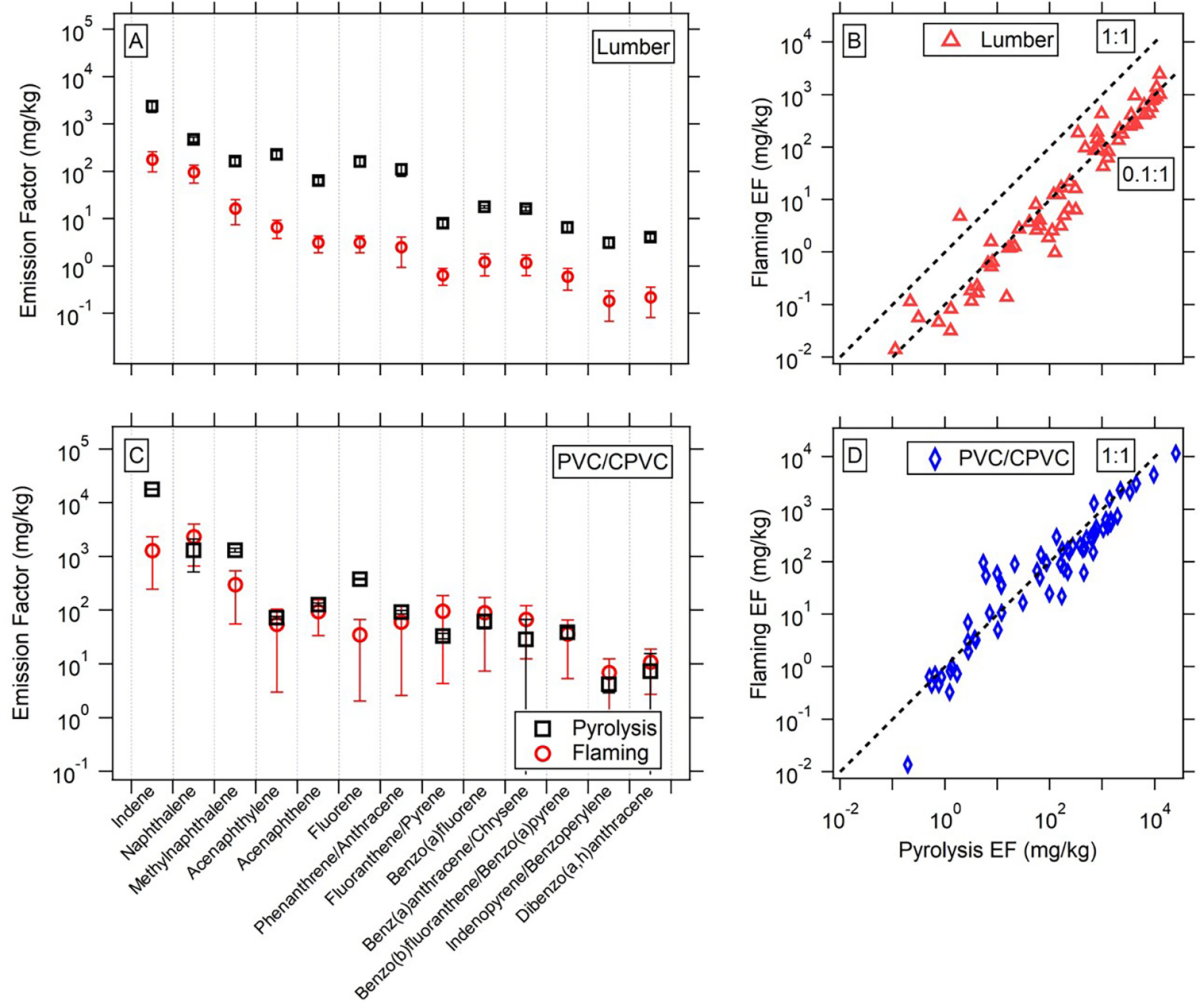
(A, C) Comparison of flaming and pyrolysis emission factors for lumber materials and plastic materials (PVC, CPVC) across a subset of VOC species. (B, D) Scatter plots of EF values for all VOCs measured in this study compared to reference lines (1:1, 0.1:1).

When we look at the same relationship for plastics (PVC, CPVC), the pattern is clear: pyrolysis and flaming of these materials yield very similar EFs for each VOC. We observe some compounds, notably several PAHs, that show higher emissions during flaming experiments (Panel C). These results indicate that the emissions from synthetic materials could have more temperature dependence as opposed to process (flaming, pyrolysis) dependence. Both processes had similar temperature ranges in these experiments (∼400–700 °C), meaning that temperature-dependent relationships would not be tested, leading to similar profiles. In future work, this relationship could be examined by looking at the temperature range more systematically across different burn conditions. There could also be formation of these specific VOCs in the flame, given the enhancements seen, but again, further experiments around these relationships are needed. These results highlight the complexity of synthetic emissions and show that for some of these structural materials, the emissions might be less related to the process they undergo and more determined by the physical structure itself or temperature. For example, the difference between the physical makeup of nylon fibers in carpet versus solid nylon jacketing around wiring could impact emissions. Other trends in material classes are shown in Figure S3. These data show that processed wood closely resembles lumber, while other materials show less of a difference between pyrolysis and burning, similar to plastics.

### Unique VOC Emissions

A range of materials is burned in WUI fires, and these data were examined to identify VOCs that may be good tracer compounds for the burning of specific materials. Such tracers could be useful in the interpretation of field data obtained downwind from or after WUI fires. The full mass spectrum for each experiment was used to create the broadest comparison possible across the full peak list. Because all VOCs in the spectra cannot be quantified, these are qualitative comparisons of the spectral signals from each experiment to highlight high-signal compounds.

In Figure 4, we compare high-resolution spectra for each material with a “biomass” spectrum. The biomass spectrum, in this case, was an average signal spectrum derived from all the lumber experiments (DFU, SYP, JS) in the relevant mode (flaming or pyrolysis). This approach allowed for internal consistency in comparisons and best simulated emissions from purely biomass wildfire materials, given these lumber materials have limited coatings or adhesives. To best compare different experiments, the spectra were corrected for differences in fuel mass and dilution factor to give a more normalized signal. Example comparisons for both flaming and pyrolysis experiments are given in Figure 4A,C. A log–log comparison was used with each spectrum. We examine the average coefficient of determination (*R* ^2^) from log comparisons for each material to identify trends in this correlation across fuels (Figure 4B,D). Lumber and processed woods generally show the highest general similarity (*R* ^2^ > 0.7), though there is still variability from burn to burn. We see less similarity for the synthetic materials.

**Figure 4:**
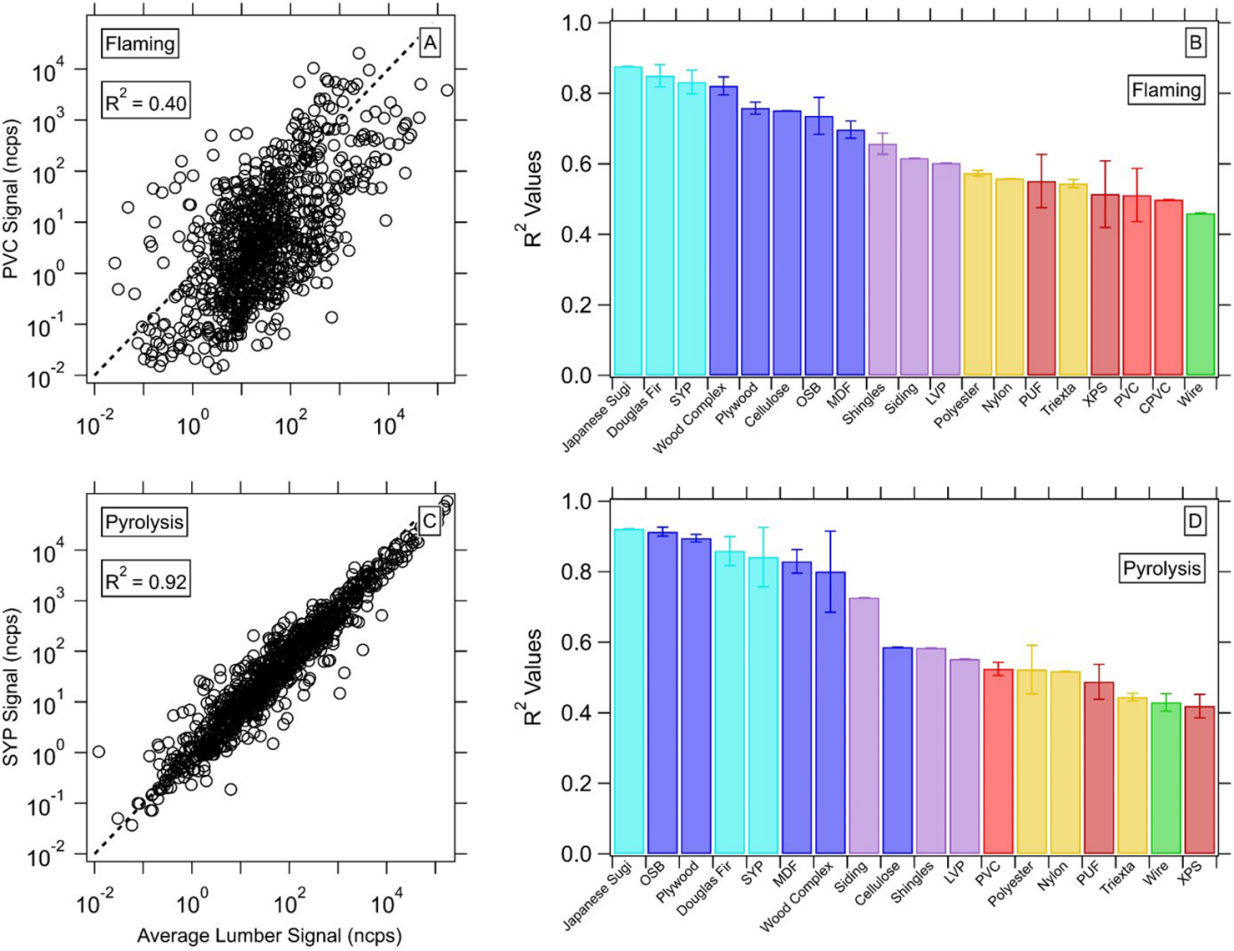
(A, C) Mass spectra comparisons between the average lumber spectrum and an example material for flaming and pyrolysis, respectively. The fit coefficient is shown for each of these examples. (B, D) Values across all materials for each material type, with error bars giving the standard deviation. The color of bars represents the general material type (light blue–lumber, blue–processed wood, yellow–carpet, green–wire, red–plastics, dark red–insulation, purple–shingles/siding/flooring).

Based on these comparisons of mass spectra, we can use residual values (eq 3) relative to a one-to-one line to see the most notable emission compounds within the peak list from each material, for example, PVC, relative to the average lumber in Figure 4A.

3 residualvalue=measuredVOCsignal−expectedvalue

The best tracers will have orders of magnitude higher emissions from one material over another. Such large differences are needed because the mass of, say, a synthetic polymer in a home can be orders of magnitude lower than the mass of lumber. We sorted the masses with potential formula compositions by their residuals. The compounds with the highest residuals for flaming and pyrolysis experiments are presented in Figure S4 and values/masses/and formulas are given in Tables S6 and S7. We see generally higher residual values for the synthetic materials than for the biomass materials, in agreement with Figure 4A.

- For PUF, we detect C_7_H_7_NOH^+^ as a high residual compound, which, when compared to the general formula for PUF, reflects the aromatic portion of the chemical formula, C_7_H_5_NO.
- In CPVC, we see C_6_Cl_2_H_4_^+^ in the flaming experiment, which could be a fragment of the general chemical monomer, C_9_Cl_7_H_11_.
- For electrical wire, we note the compound C_6_H_11_NOH^+^ is present among the highest ten residuals and matches the base nylon formula, C_6_H_11_NO.
- However, we also see less expected compounds, such as C_8_H_25_O_4_Si_4_^+^ in PUF and C_4_H_7_S_2_Si^+^ in CPVC, which do not closely match the base chemical formula, indicating a flaming product or production additive in the material.
- There are also interesting outliers, particularly with the cellulose and wood complex experiments in the pyrolysis graph, showing high residuals of halogenated compounds (C_2_Cl_2_H_2_^+^, CH_4_Br^+^) compared to the reference spectrum.

It should be noted that these assignments are tentative, especially around higher mass values, and we have included high-resolution mass values in addition to formulas as a potential resource moving forward for identifying what compounds might act as tracers for each of these materials and subsequent emissions. Additionally, as the amounts of materials are increased or mixtures of materials undergo burning, it could shift VOC emissions. Especially halogenated compounds, which can have faster secondary chemistry, could react in mixtures to form different compounds, shifting the emissions and subsequent tracer compounds.

### Structural Emission Estimates

Using EF data collected in this work and an estimate for the composition of materials used in a typical home (Table S4), we calculated emissions amounts for the flaming combustion of a representative single-family home.^25,31^ The composition was informed by material distributions presented in the report The *Chemistry of Fires at the Wildland–Urban Interface* (2022) and by taking into account the materials used in this study.^25^ This analysis is limited to only one profile comparison and could be enhanced by considering a wider range of material types in future work. It may also shift as different materials are considered beyond those looked in this study, varying both the composition and age of materials; this is still a useful emissions profile, though, for calculating the impacts of WUI fires and highlighting which VOCs may be enhanced over more traditional fire scenarios. We assume that the emissions of materials will be consistent when scaled to larger masses, as the emissions could vary with larger and mixed blocks of materials as opposed to the small single amounts used here.^32^

The emission composition of this synthesized home was compared to the emission composition of the same mass of structural Douglas Fir (Figure 5A) derived from this study. This highlights which VOC compounds might be elevated due to the more synthetic materials used in a home and which ones are largely derived from the lumber present. Figure 5A shows that several compounds show elevated emission amounts in the mixed material profile. Notably, aromatics, such as benzene and styrene, and PAH compounds, such as naphthalene, are enhanced. Additionally, we see enhancements in nitrogen compounds, such as acetonitrile, pyridine, and benzonitrile, derived from the high nitrogen content in materials such as polyurethane and nylon coatings (Figure S5). The enhancements seen here from materials that represent only a fraction of the total mass of a home, such as carpet, show that the higher EFs can compensate for the lower mass fraction. However, it should be noted that many compounds show similar values, indicating the difficulty of differentiating WUI smoke from traditional biomass smoke emissions, which is compounded by the fact that a large portion of the fuel in some WUI fires is vegetation.

**Figure 5:**
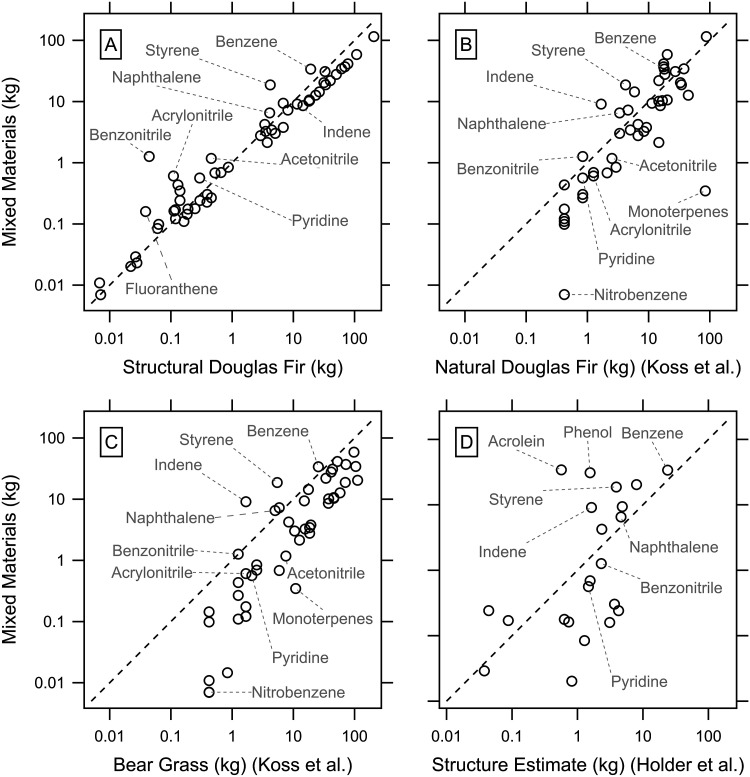
Mass of VOC emissions estimated from a flaming burn of 42000 kg of material for various compositions. The “mixed material” distribution represents a makeup of materials reflecting a single-family home (Table S5). Dashed lines indicate one-to-one lines in each panel. (A) Comparison of the mixed material to a profile of structural Douglas Fir wood from this study. (B, C) Comparison of the profile to a profile of overlapping VOCs from natural Douglas Fir and Bear Grass, respectively, taken from Koss et al.^16^ (D) VOC overlap from the structural subset values in Holder et al.^23^

We also examined a comparison of the calculated whole-house burning emissions to emissions from the same mass of natural Douglas Fir as published by Koss et al. (Figure 5B).^16^ This comparison is different, as natural Douglas Fir includes bark and needles compared to just heartwood. Higher emissions of styrene and indene are still noted in the structural profile compared to the natural wood. In contrast, emissions of nitrobenzene and monoterpenes are elevated compared to those of Douglas Fir. This is likely due to the needles, branches, and bark of trees having higher nitrogen and monoterpene content than the structural Douglas Fir.^33^ We also compare the whole-house profile versus the emission profile of bear grass from Koss et al. (Figure 5C) to account for grassland fires that may transition to the WUI area, as with the Marshall Fire in 2021 and the Lahaina Fire in 2023.^34,35^ The EF values for grasses were found to be higher than those for woods in the Koss et al.^16^ study, meaning that in this comparison, there are fewer compounds with higher values for the structure, though styrene and indene continue to stand out.

In Figure 5D, we compare overlapping VOCs from this study to values determined for structural emissions in the study by Holder et al.^23^ Here, we see a greater spread of EF values, likely due to differences in structural materials or makeup, which have a high impact on the values. These differences emphasize the complexity of structural emissions and the difficulty of creating an overall emission picture, given the breadth of materials and different emission mechanisms.

The enhancement of PAH values is notable due to work showing that these compounds, in particular, can linger in indoor spaces for longer time scales.^12^ These enhancements are important when placed in the context of large wildland–urban interface fires, such as those in Los Angeles in early 2025, which burned over 18,000 structures, where exposure to certain VOC compounds could be magnified for those around the fire. The comparison across all of these profiles speaks to the difficulty in discerning structural smoke from biomass smoke, especially if atmospheric aging occurs. However, we do find evidence of the enhancement of some compounds in WUI fires relative to biomass fires.

## Supplementary Material





## Notes

Overview of experimental materials and experiment breakdown, data sheets with concentrations and EF values, data sheets with spectra residuals/masses/formulas, intra- and interexperimental comparisons, and further figures on mass spectra comparisons and EF results (PDF)
Table S3, Concentrations of VOCs (ppbv); Table S4, Emmission factors (Efs) (mg/kg); Table S6, Masses, residuals, formulas for flaming experiments; Table S7, Masses, residuals, formulas for pyrolysis experiments (XLSX)
