# Supplementary material for: Laboratory Analysis of VOC Emissions from Structural Materials in Wildland–Urban Interface Fires
Source: Environ Sci Technol. 2026 Feb 20;60(9):7251–9. doi: 10.1021/acs.est.5c11276 (PMC12980821; doi:10.1021/acs.est.5c11276)
Supplement: Supplementary file 1 [file es5c11276_si_001.pdf]

Supporting Information for Laboratory Analysis of VOC Emissions from Structural Materials in Wildland-Urban Interface Fires

William Dresser[1,2], Kevin Ridgway[3], Anna Helfrich[3], Christian L'Orange[3], Shantanu Jathar[3], Joost de Gouw[1,2]*

[1]Department of Chemistry, University of Colorado, Boulder, CO, USA, 80309

[2]Cooperative Institute for Research in Environmental Science, University of Colorado/NOAA, Boulder, CO, USA, 80309

[3]Department of Mechanical Engineering, Colorado State University, Fort Collins, CO, USA, 80523

*Corresponding Author: Joost.deGouw@colorado.edu

Supplementary Information

This Supplementary Information (SI) file and the attached spreadsheet files support the paper by providing background information into the experimental set-up and methods. It includes 8 pages, 5 Figures, and 6 Tables. In addition, it contains comprehensive tables on emission factor (EF) and residual values and contains further data analysis on experimental validation, mass spectra residuals, and EF values.

Table of Contents

Table S1. Material List, Experiment Type, and Experiment Number……………………………………S2
Table S2. Overview of Calibration VOCs……………………………………………………..……S3
Figure S1. Comparison of EF calculation Methods……………………………………………………S4
Figure S2. Comparison of online versus offline VOC results ……………………………...…………S5
Figure S3. Flaming versus pyrolysis EF values for material types………………………………………S6
Figure S4. Residual values for mass spectra fit against biomass average spectrum……...…...…………S7
Table S5. Overview of material percentages for structure profile……………………..………………S8
Figure S5. Summary of EF values of nitrogen species for flaming experiments………...………………S9

Table S1: Summary of Fuels tested, category of each fuel, and flaming/pyrolysis experiments for each fuel.

**Fuel Type**	**Category**	**Flaming Experiments**	**Pyrolysis Experiments**
Douglas Fir (DF)	Lumber	3	3
Southern Yellow Pine (SYP)	Lumber	3	4
Japanese Sugi	Lumber	1	1
Oriented Strand Board (OSB)	Processed Wood	3	3
Medium-Density Fiberboard(MDF)	Processed Wood	2	2
Plywood	Processed Wood	2	3
Cellulose	Processed Wood	1	1
Wood Complex*	Processed Wood	2	2
Extruded Polystyrene(XPS)	Insulation	2	2
Polyurethane Foam (PUF)	Insulation	2	2
Polyvinyl Chloride (PVC)	Plastic/Siding	2	2
Chlorinated Polyvinyl Chloride (CPVC)	Plastic/Siding	1	1
Asphalt Shingles	Shingles	1	2
Nylon	Carpet	1	1
Triexta	Carpet	2	2
Polyester	Carpet	3	3
Cement Fiber Siding (Siding)	Siding	1	1
Luxury Vinyl Plank (LVP)	Flooring	1	1
12/14-gauge wire	Wire	2	2
Blanks	–	2	3

**Wood Complex Make-up:37±2% SYP, 37±4% DF, 5±1% MDF, 13±3% OSB, 7±1% Plywood*

Table S2: Overview of calibration species and concentrations

Compound Name	Concentration (ppbv) ± 5%
Acetaldehyde	1026
Acetonitrile	1064
Acetone	993
Acrylonitrile	996
Isoprene	992
Methyl Ethyl Ketone	975
Benzene	1014
Toluene	989
M-Xylene	1005
Alpha-Pinene	1007
1,2,4-Trimethylbenzene	997
Beta-Caryophyllene	99.7

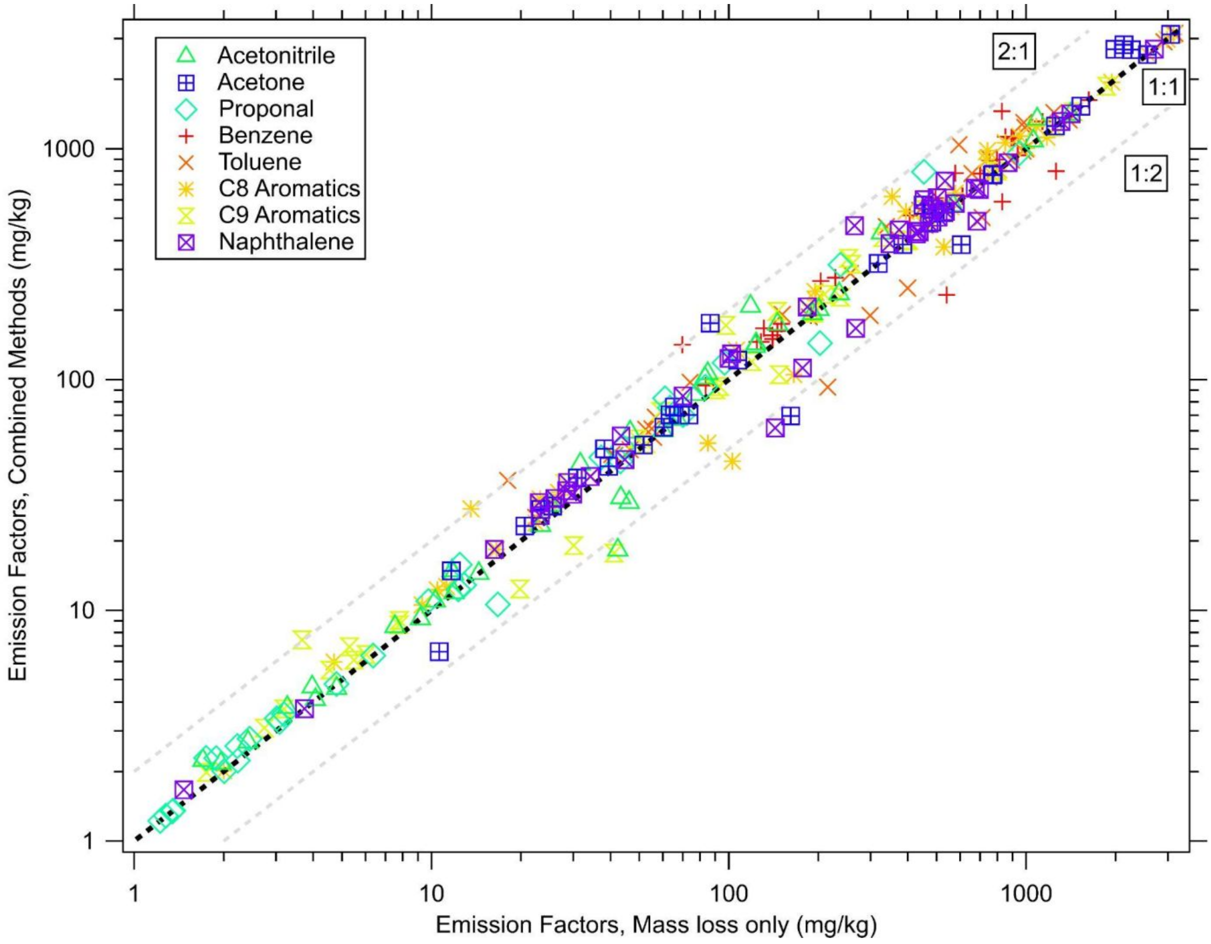

Figure S1: Comparison of emission factors calculated with the method outlined in Equation 1 and Equation 2 on the y-axis versus values calculated only using the mass loss approximation equation (Equation 2) on the x-axis.

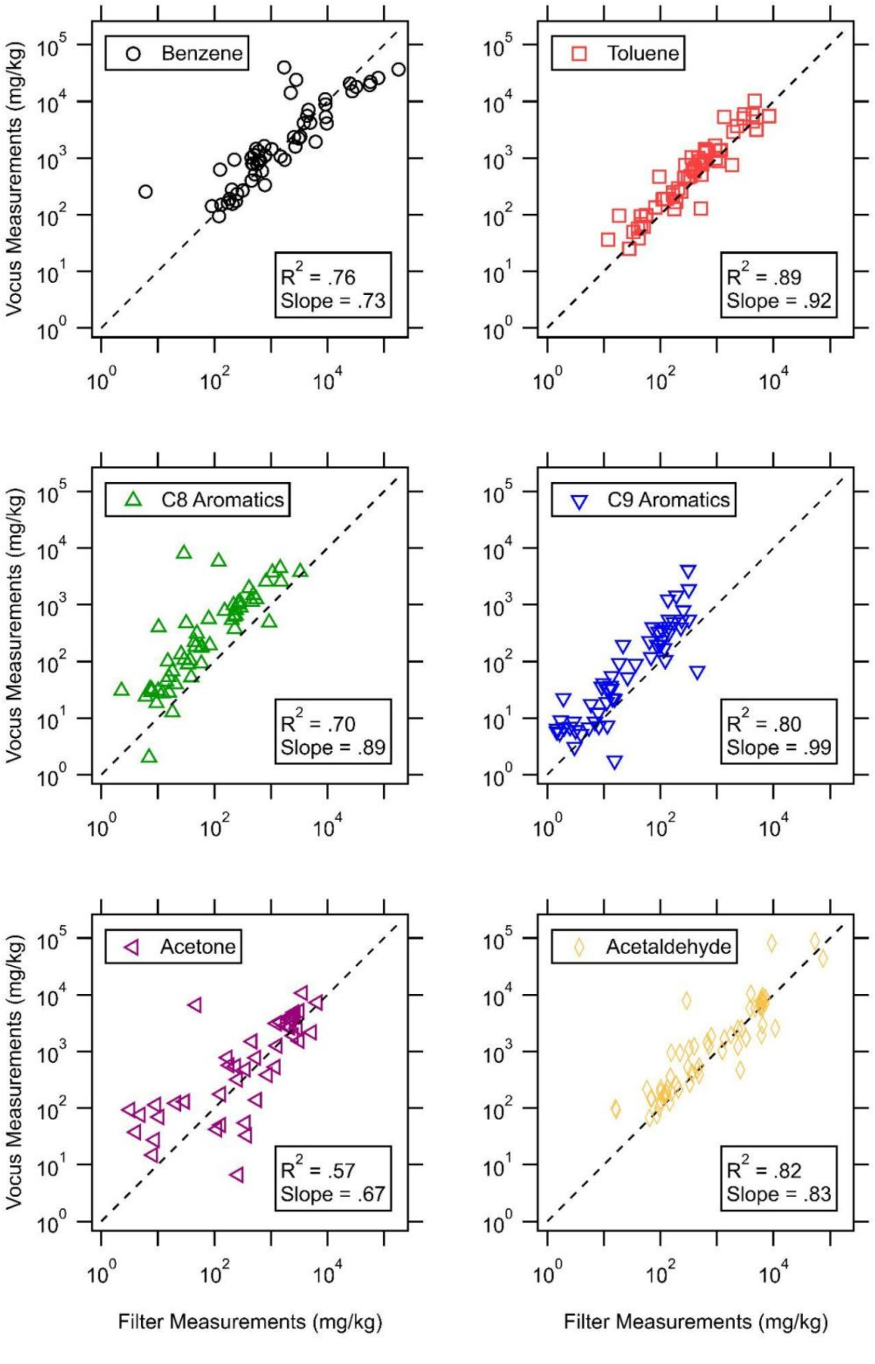

Figure S2: Comparison of emission factors from this work against those collected on filters and analyzed offline by GC-MS. Each panel shows values for one VOC across all burns with data from these data on the y-axis and comparison data from filter collection on x-axis. One-to-one lines are included in each panel.

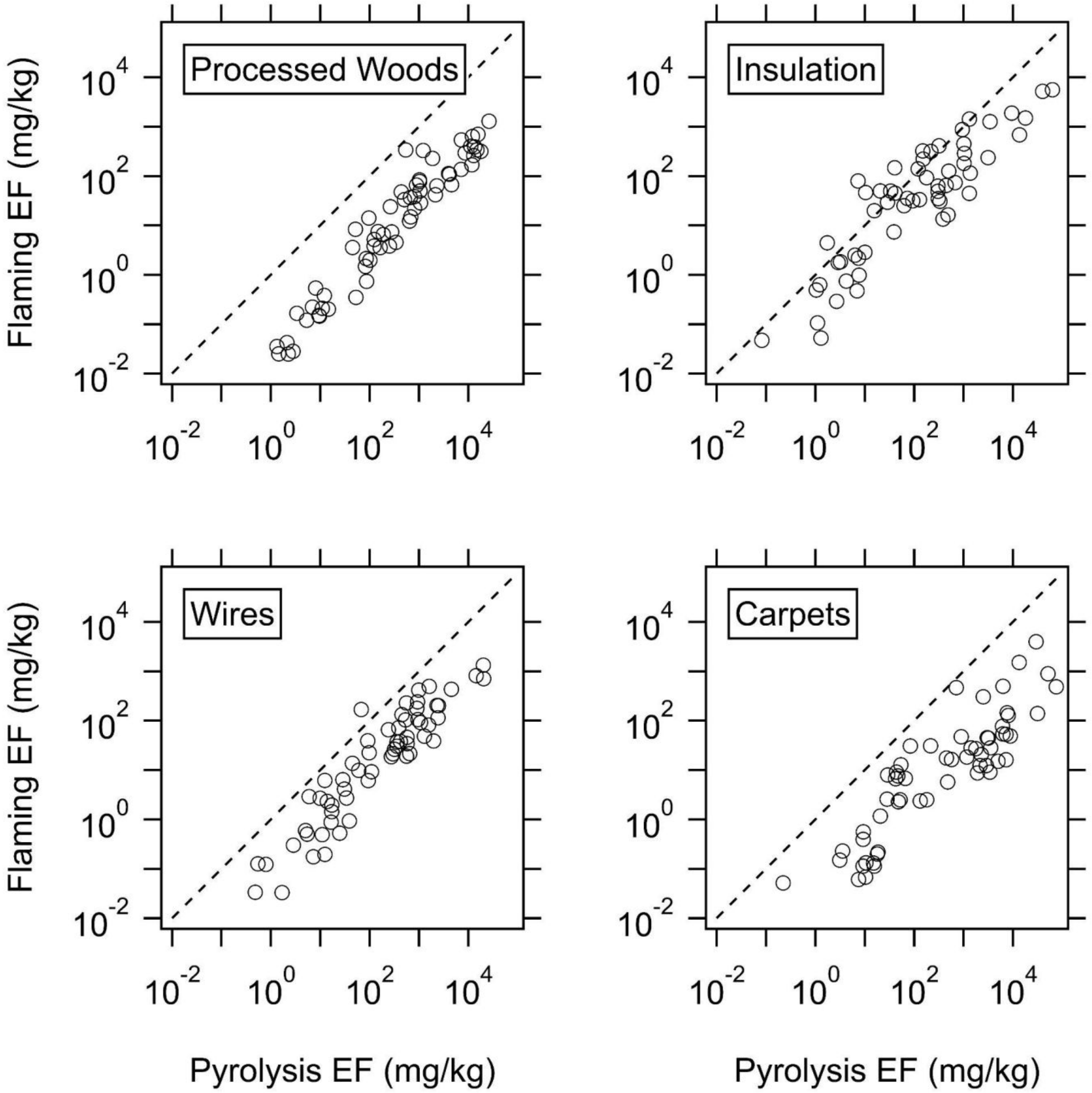

Figure S3: Comparisons of VOCs emission factors from flaming and pyrolysis experiments across several classes of materials. One to one line shown by a dashed line in each panel.

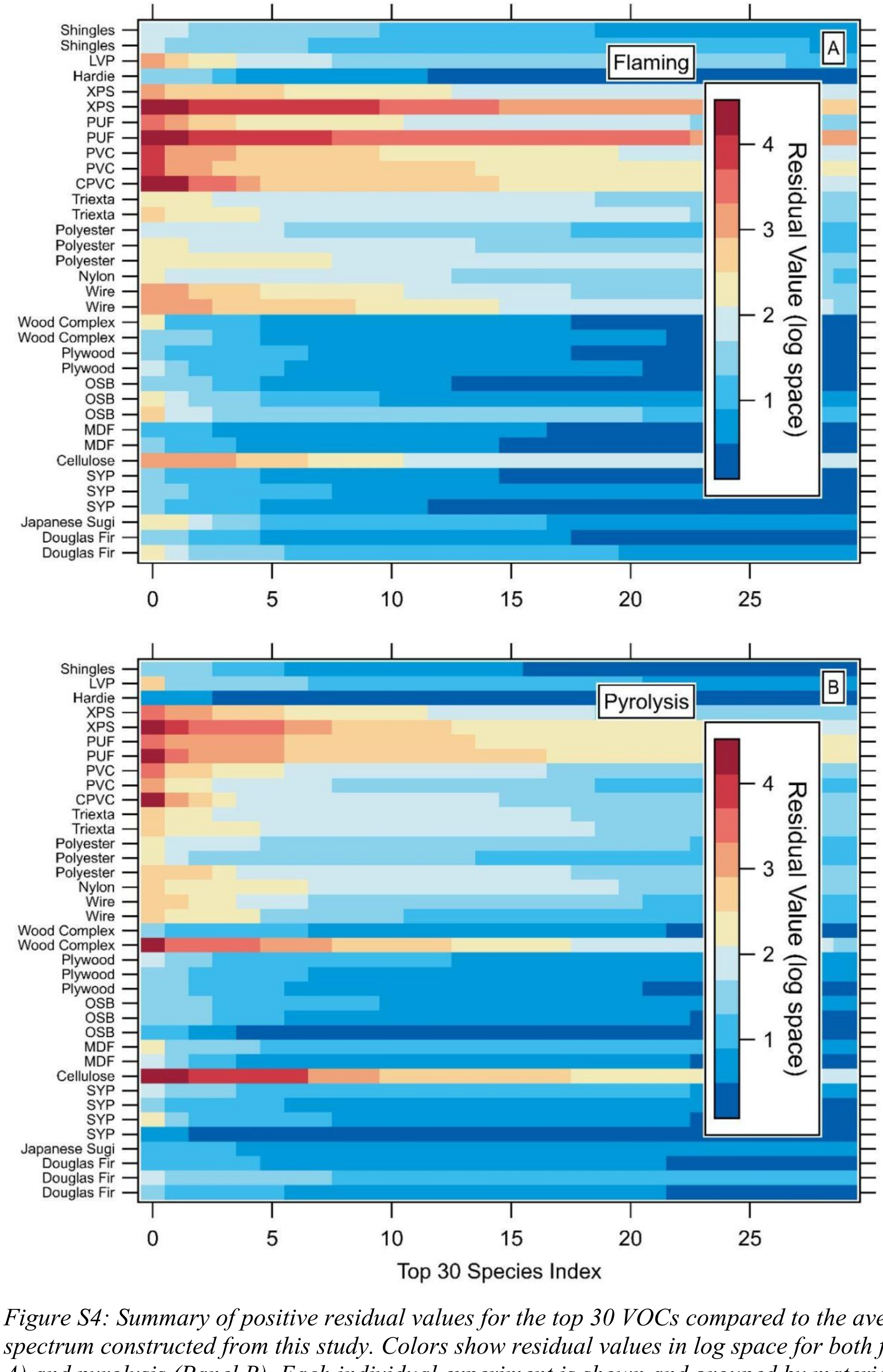

Figure S4: Summary of positive residual values for the top 30 VOCs compared to the average lumber spectrum constructed from this study. Colors show residual values in log space for both flaming (Panel A) and pyrolysis (Panel B). Each individual experiment is shown and grouped by material category.

Table S5: Summary of "mixed material" profile make-up used in Figure 5.

**Material**	**Percentage (by mass)**
Douglas Fir	50%
SYP	10%
Plywood	10%
MDF	10%
OSB	10%
Wire	1%
Triexta	1%
Polyester	2%
Nylon	1%
PVC	1%
CPVC	1%
XPS	1%
PUF	1%
Shingles	1%

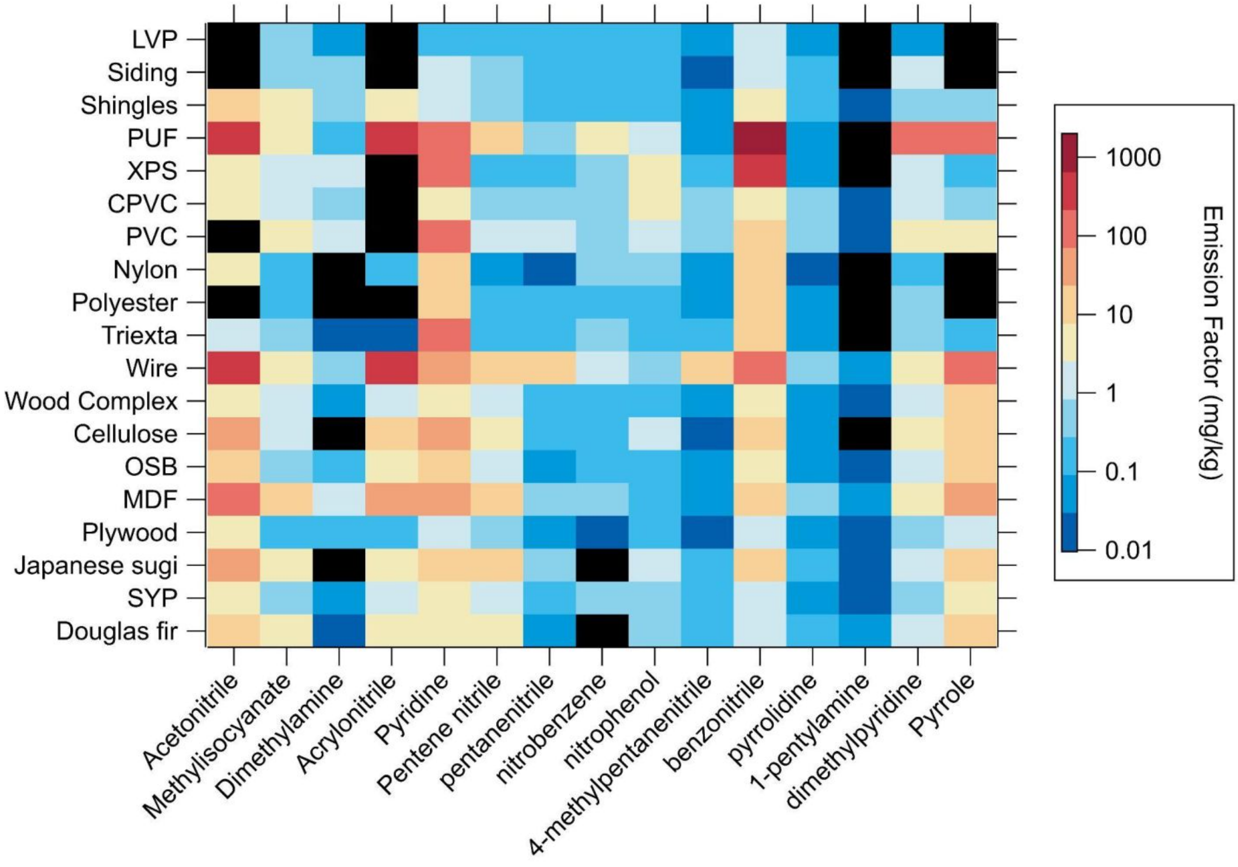

Figure S5: Summary of EF values for nitrogen species quantified in this experiment. Black squares represent values that were not quantifiable after background corrections.
